# Baseline laboratory parameters for preliminary diagnosis of COVID-19 among children: a cross-sectional study

**DOI:** 10.1590/1516-3180.2021.0634.R1.05012022

**Published:** 2022-08-12

**Authors:** Dejan Dobrijević, Jasmina Katanić, Maša Todorović, Biljana Vučković

**Affiliations:** I MD. Clinical Biochemistry Resident and Teaching Assistant, Department of Biochemistry, Medical Faculty, University of Novi Sad, Serbia; and Physician, Department of Laboratory Diagnostics, Institute for Child and Youth Health Care of Vojvodina, Novi Sad, Serbia.; II MD, PhD. Medical Biochemist, Department of Laboratory Diagnostics, Institute for Child and Youth Health Care of Vojvodina, Novi Sad, Serbia; and Assistant Professor, Department of Biochemistry, Medical Faculty, University of Novi Sad, Serbia.; III MD. Laboratory Medicine Resident, Department of Laboratory Medicine, Clinical Center of Vojvodina, Novi Sad, Serbia; and Teaching Assistant, Department of Pathophysiology and Laboratory Medicine, Medical Faculty, University of Novi Sad, Serbia.; IV MD, PhD. Internal Medicine Physician, Department of Laboratory Medicine, Clinical Center of Vojvodina, Novi Sad, Serbia; and Associate Professor, Department of Pathophysiology and Laboratory Medicine, Medical Faculty, University of Novi Sad, Serbia.

**Keywords:** COVID-19, SARS-CoV-2, Child, Biomarkers, Children with COVID, Laboratory diagnosis, Control group selection

## Abstract

**BACKGROUND:**

Clinical judgment of initial baseline laboratory tests plays an important role in triage and preliminary diagnosis among coronavirus disease 2019 (COVID-19) patients.

**OBJECTIVES:**

To determine the differences in laboratory parameters between COVID-19 and COVID-like patients, and between COVID-19 and healthy children. Additionally, to ascertain whether healthy children or patients with COVID-like symptoms would form a better control group.

**DESIGN AND SETTING:**

Cross-sectional study at the Institute for Child and Youth Health Care of Vojvodina, Novi Sad, Serbia.

**METHODS:**

A retrospective study was conducted on 42 pediatric patients of both sexes with COVID-19. Hematological parameters (white blood cell count, absolute lymphocyte count and platelet count) and biochemical parameters (natremia, kalemia, chloremia, aspartate aminotransferase [AST], alanine aminotransferase [ALT], lactate dehydrogenase [LDH] and C-reactive protein [CRP]) were collected. The first control group was formed by 80 healthy children and the second control group was formed by 55 pediatric patients with COVID-like symptoms.

**RESULTS:**

Leukocytosis, lymphopenia, thrombocytosis, elevated systemic inflammatory index and neutrophil-lymphocyte ratio, hyponatremia, hypochloremia and elevated levels of AST, ALT, LDH and CRP were present in COVID patients, in comparison with healthy controls, while in comparison with COVID-like controls only lymphopenia was determined.

**CONCLUSIONS:**

The presence of leukocytosis, lymphopenia, thrombocytosis, elevated systemic inflammatory index and neutrophil-lymphocyte ratio, hyponatremia, hypochloremia and elevated levels of AST, ALT, LDH and CRP may help healthcare providers in early identification of COVID-19 patients. Healthy controls were superior to COVID-like controls since they provided better insight into the laboratory characteristics of children with novel betacoronavirus (SARS-CoV-2) infection.

## INTRODUCTION

The ongoing pandemic of coronavirus disease 2019 (COVID-19), caused by a novel betacoronavirus (SARS-CoV-2), is the number-one public health emergency, with more than 192 million cases worldwide.^1,2^ In the Republic of Serbia, the outbreak is still ongoing with more than 941,000 registered cases so far. At the moment, the epidemiological situation is stable with a further decreasing trend in the COVID-19 incidence rate in all parts of the country.^3^ Pneumonia was the initial clinical sign of COVID-19 that enabled case detection. Asymptomatic infections are common, especially among young children, and play an important role in spreading the disease.^4,5^

Making timely diagnoses is of paramount importance for appropriate management, taking into account the global epidemiology and mortality risk of COVID-19. The real-time polymerase chain reaction (RT-PCR) is considered to be the gold standard for identification of SARS-CoV-2. In addition to molecular genome sequencing, rapid antigen and serological tests are also performed in many countries.^6^ However, human resource and laboratory capacities are often insufficient to ensure massive and prompt diagnostics. Since the time taken to present the results from etiological RT-PCR may be prolonged, clinical judgment of the initial baseline laboratory tests plays an important role in triage and preliminary diagnosis.

Several laboratory parameters have been recommended for distinguishing SARS-CoV-2-positive patients from patients with COVID-like symptoms.^5,6^ According to the official guidelines (the WHO interim guidelines and the guidelines of the National Health Commission of China for COVID-19, 5^th^ edition), white blood cell counts and lymphocyte counts are significant for early diagnosis. Thrombocytosis is another common laboratory finding.^7^ Hematological indices such as the systemic inflammatory index (SII), neutrophil-lymphocyte ratio (NLR) and platelet-lymphocyte ratio (PLR) are useful biomarkers for assessments of disease severity and prognosis among patients with pneumonia.^8^ Electrolyte disturbances such as hyponatremia, hypokalemia and hypochloremia have been corelated with COVID-19 infection.^9^ Even in mild cases of COVID-19, hepatic transaminase levels (alanine aminotransferase [ALT] and aspartate aminotransferase [AST]) may be elevated due to transient liver damage.^10^ Lactate dehydrogenase (LDH) is an enzyme associated with tissue damage and is a biomarker of interest in COVID-19 patients.^11,12^ C-reactive protein (CRP) is an important clinical biomarker of inflammation and infection. Altered CRP levels may be linked to the degree of disease severity among COVID-19 patients.^13^

All of these data were primarily documented from adult cases. The incidence of manifest COVID-19 in the pediatric population is significantly lower than the incidence of infected adults.^8^ Therefore, information about hematological and biochemical parameter alterations in children with COVID-19 is very limited worldwide. Furthermore, the interpretation of these results varies to a significant extent.^8,14^

## OBJECTIVE

The objective of this study was to determine the differences in laboratory parameters between COVID-19 and COVID-like patients, and between COVID-19 patients and healthy children. Additionally, we aimed to find out whether healthy children or patients with COVID-like symptoms would form a better control group.

## METHODS

A retrospective study was conducted on 42 pediatric patients of both sexes with COVID-19. All of these patients were admitted to the confirmed-infection isolation wards at the Institute for Child and Youth Health Care of Vojvodina between April 2020 and January 2021. The sample size was not calculated because we had 42 COVID cases in total within the abovementioned period and we included all of them in the study. Occurrences of SARS-CoV-2 infection were confirmed through RT-PCR, performed on nasopharyngeal and throat swab specimens from the patients.

The hematological and biochemical findings from blood samples collected on the day of admission were recorded. The patients did not receive any therapy before blood collection. The hematological values were tested using the Advia 2120 hematology analyzer (Siemens Healthcare, Germany), for complete blood counts with a differential white blood cell count. The following hematological results were collected: white blood cell count, absolute lymphocyte count and platelet count. Additionally, SII, NLR and PLR were calculated. The biochemical values were tested using the AU 480 chemistry analyzer (Beckman Coulter, Switzerland). The following biochemical results were collected: natremia, kalemia, chloremia, AST, ALT, LDH and CRP.

For comparative analyses, two control groups of age and sex-matched SARS-CoV-2-negative patients were enrolled in the study. The first control group was formed by 80 children who were healthy at that moment and had come to our institution for their regular check-ups. A total of 55 pediatric patients with COVID-like symptoms formed the second control group. Patients with malignancy were excluded from our analysis. All the controls were tested negative for COVID-19 prior to admission by means of a rapid test for qualitative detection of SARS-CoV-2 antigen (Panbio COVID-19 Ag Rapid Test Device, Abbott) and/or an immunochromatographic IgM/IgG antibody assay (Innovita COVID-19 immunoglobulin M [IgM]/immunoglobulin G [IgG] rapid test). Informed consent was waived because of the retrospective nature of the study, and the analyses used anonymous laboratory data.

Statistical analyses (descriptive and inferential) were performed using the Statistical Package for the Social Sciences (SPSS version 26.0) software (IBM Corporation, Armonk, New York, United States). This study was approved by the ethics committee of the Institute for Child and Youth Health Care of Vojvodina (December 23, 2020; no. 4881-2).

## RESULTS

Between April 2020 and January 2021, a total of 42 pediatric cases of COVID-19 infection were admitted to the Institute for Child and Youth Health Care of Vojvodina. These patients’ average age was 5.24 ± 6.04 years. The female share of the group was 57.1%, with an average age of 6.12 ± 6.54 years; and 42.9% of the group were males with an average age of 4.07 ± 5.25 years. The first control group consisted of 80 healthy children with an average age of 5.84 ± 6.12 years. The female share of this group was 41.3%, with an average age of 6.63 ± 6.46 years; and 58.7% of the group were males with an average age of 5.28 ± 5.89 years. The second control group was formed by 55 children with COVID-like symptoms, with an average age of 5.39 ± 5.81 years. The female share of this group was 49.1%, with an average age of 5.38 ± 5.98 years; and 50.9% of the group were males with an average age of 5.40 ± 5.73 years. The composition of the groups according to age and sex is presented in Table 1.

**Table 1.** Age-sex structure of coronavirus disease 2019 (COVID-19) group, healthy controls and COVID-like controls

Patient features		Total (n = 177)	COVID-19 group (n = 42)	Healthy controls (n = 80)	COVID-like controls (n = 55)
**Gender**	Female	84 (47.5%)	24 (57.1%)	33 (41.3%)	27 (49.1%)
	Male	93 (52.5%)	18 (42.9%)	47 (58.7%)	28 (50.9%)
**Age (years)** ^*^	General	5.56 ± 5.98	5.24 ± 6.04	5.84 ± 6.12	5.39 ± 5.81
		3.0 (0.44-11.0)	5.50 (0.25-10.0)	3.0 (0.74-11.0)	3.0 (0.44-12.0)
	Female	6.08 ± 6.29	6.12 ± 6.54	6.63 ± 6.46	5.38 ± 5.98
		4.0 (0.46-12.75)	6.00 (1.25-10.0)	4.0 (0.83-13.5)	3.5 (0.54-13.0)
	Male	5.08 ± 5.68	4.07 ± 5.25	5.28 ± 5.89	5.40 ± 5.73
		3.0 (0.44-10.0)	5.00 (0.23-10.0)	3.0 (0.5-10.0)	3.0 (0.43-12.0)

* Mean ± standard deviation/median (interquartile range: Q1–Q3).

Binary logistic regression was used to determine that age was not a risk factor for COVID-19 (P = 0.601; odds ratio, OR: 1.017; 95% confidence interval, CI: 0.955-1.083). No confounding factors were identified.

Leukocytosis, lymphopenia and thrombocytosis, elevated SII and NLR were present in COVID patients, in comparison with healthy controls; while in comparison with the COVID-like controls, only lymphopenia was determined (Tables 2 and 3). No significant differences in biochemical parameters between the COVID and COVID-like groups were found. On the other hand, we determined hyponatremia, hypochloremia and elevated levels of AST, ALT, LDH and CRP in the COVID-positive patients, in comparison with the healthy controls (Table 4).

**Table 2.** Hematological characteristics of the coronavirus disease 2019 (COVID-19) group, healthy controls and COVID-like controls^*^

Characteristic	COVID-19 group (group 1)	Healthy controls (group 2)	COVID-like controls (group 3)	P value^†^	
				1 versus 2	1 versus 3
Leukocytosis	12 (28.6%)	0	14 (26.9%)	**P < 0.001**	P = 0.541
Lymphopenia	7 (16.7%)	0	1 (1.9%)	**P < 0.001**	**P = 0.025**
Thrombocytosis	9 (21.4%)	2 (2.5%)	14 (26.9%)	**P < 0.001**	P = 0.723

* Values are n (% within group); ^†^chi-square test and Fisher’s exact test. Values in bold are statistically significant.

**Table 3.** Hematological indices/ratios in the coronavirus disease 2019 (COVID-19) group, healthy controls and COVID-like controls^*^

Hematological indices/ratios	COVID-19 group (group 1)	Healthy controls (group 2)	COVID-like controls (group 3)	P value^†^	
				1 versus 2	1 versus 3
SII	1069.62	475.83	746.44	**P = 0.008**	P = 0.305
NLR	3.46	1.86	2.11	**P = 0.007**	P = 0.100
PLR	126.89	109.73	114.18	P = 0.254	P = 0.546

* Values are represented as mean values; ^†^independent-samples t test. Values in bold are statistically significant. SII = systemic inflammatory index; NLR = neutrophil-lymphocyte ratio; PLR = platelet-lymphocyte ratio.

**Table 4.** Biochemical characteristics of the coronavirus disease 2019 (COVID-19) group, healthy controls and COVID-like controls^*^

Biochemical parameter	COVID-19 group (group 1)	Healthy controls (group 2)	COVID-like controls (group 3)	P value^†^	
				1 versus 2	1 versus 3
Natremia (mmol/l)	136.43	141.28	135.48	**P < 0.001**	P = 0.211
Kalemia (mmol/l)	4.72	4.69	4.52	P = 0.785	P = 0.293
Chloremia (mmol/l)	102.15	103.83	100.42	**P = 0.017**	P = 0.549
AST (μkat/l)	0.94	0.47	0.88	**P < 0.001**	P = 0.832
ALT (μkat/l)	0.71	0.33	0.68	**P = 0.002**	P = 0.907
LDH (μkat/l)	5.51	3.74	4.69	**P < 0.001**	P = 0.155
CRP (mg/l)	38.63	1.15	53.82	**P < 0.001**	P = 0.280

* Values are represented as mean values; ^†^independent-samples t test. Values in bold are statistically significant. AST = aspartate aminotransferase; ALT = alanine aminotransferase; LDH = lactate dehydrogenase; CRP = C-reactive protein.

**Figure 1.**
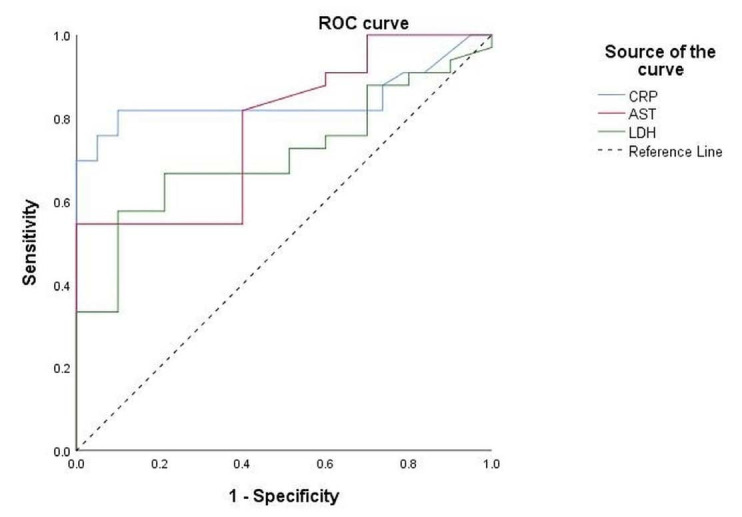
Receiver operating characteristic curve for the most significant laboratory parameters in predicting coronavirus disease 2019 (COVID-19)-positive patients.

Furthermore, statistically significant laboratory parameters were studied through receiver operating characteristic (ROC) analyses. Only laboratory markers with an area under the curve (AUC) above 0.7 were considered acceptable for analyzing the exact cutoff value: CRP (AUC: 0.842), AST (AUC: 0.779) and LDH (AUC: 0.712) (Figure 1). The cutoff point for CRP values was set at 2.1 mg/l (P < 0.001; sensitivity: 81.8%, specificity 90%; 95% CI: 0.773–0.951). The cutoff point for AST values was set at 0.49 μkat/l (P < 0.001; sensitivity: 81.8%, specificity 60%; 95% CI: 0.681–0.877). The cutoff point for LDH values was set at 4.22 μkat/l (P < 0.001; sensitivity: 66.7%, specificity 78.7%; 95% CI: 0.591–0.832).

ROC = receiver operating characteristic; CRP = C-reactive protein; AST = aspartate aminotransferase; LDH = lactate dehydrogenase.

Additionally, we analyzed the differences between the COVID-19 group and the healthy children according to the cutoff values for CRP, AST and LDH. These differences were statistically significant for all three parameters (Table 5).

**Table 5.** Comparison of laboratory parameters according to their cutoff points^*^

Parameter	COVID-19 patients	Healthy controls	P value^†^
CRP ≥ 2.1 mg/l	32 (76.2%)	8 (10.0%)	**< 0.001**
AST ≥ 0.49 μkat/l	29 (69.0%)	31 (38.8%)	**< 0.001**
LDH ≥ 4.22 μkat/l	22 (52.4%)	17 (21.3%)	**< 0.001**

* Values are n (% within group); ^†^chi-square test. Values in bold are statistically significant. CRP = C-reactive protein; AST = aspartate aminotransferase; LDH = lactate dehydrogenase.

## DISCUSSION

Considering the global epidemiology and alarming severity of COVID-19 infection, well-timed diagnostics are crucial.^15^ The turnaround time for RT-PCR results is supposed to be very quick, but the lack of human and laboratory resources in many countries has led to delays in SARS-CoV-2 confirmation. During this period, baseline hematological and biochemical analyses are essential for enabling a clinical judgement when COVID-19 infection is suspected.^15,16^

In the initial analyses, in which patients were compared with healthy controls, we observed leukocytosis, lymphopenia, thrombocytosis, elevated SII and NLR, hyponatremia, hypochloremia and elevated levels of AST, ALT, LDH and CRP. However, comparison of patients with COVID-19 and patients with COVID-like symptoms showed that only lymphopenia might play an important role in distinguishing patients within these two groups. In a review paper published by Lippi et al., the most common laboratory findings were leukocytosis, lymphopenia, thrombocytopenia and elevated levels of AST, ALT, LDH and CRP.^17^ Cai et al. reported occurrences of leukocytosis, lymphocytosis, thrombocytosis and elevated levels of AST, ALT, LDH and CRP.^18^ Furthermore, the laboratory findings in a study conducted by Wang et al. were leukocytosis, lymphocytosis, thrombocytosis and elevated levels of AST, ALT and CRP, while the levels of LDH were within the reference range.^19^ Thus, it can be said that the laboratory findings from different clinical and research centers vary to a significant extent.

Several factors may have influenced this discrepancy in the laboratory results. In the first place, the medical community and general public have had a constant need for new information about the COVID-19 pandemic, and this has been paramount. The methodological quality of published reports has been lower than that of similar studies published prior to the pandemic.^20^ Furthermore, this need to disseminate information promptly has been forcing researchers to opt for simpler study designs. Even in some major journals, many observational studies have been published without control groups.^21^

Additionally, selecting an appropriate comparison group is crucial. Use of more than one control group has often been discussed in observational studies. When healthy controls are used, they are expected to show any laboratory distinction between COVID-19 patients and healthy children. On the other hand, when patients with COVID-like symptoms are used as controls, it is possible to determine which laboratory parameters might be specific for SARS-CoV-2 infection, regardless of the symptoms.^22^

In our study, no specific laboratory parameters were determined. Firstly, there were no COVID-specific parameters among the baseline hematological and biochemical analyses; and secondly, there are a lot of common viral infections among children, which may present with similar laboratory findings.^22,23^ Therefore, healthy children formed a more convenient control group in our study, given that they showed us various alterations in laboratory parameters such as leukocytosis, lymphopenia, thrombocytosis, elevated SII and NLR, hyponatremia, hypochloremia and elevated levels of AST, ALT, LDH and CRP.

Lymphopenia is common in acute illness, when T-lymphocytes and NK-cells become exhausted and their counts start to decrease.^24^ Thrombocytosis in children with viral infection of the lower respiratory tract is a reactive phenomenon and does not indicate a severe clinical course.^25^

Elevated SII and NLR are indicators of inflammation associated with a dismal outcome among adult COVID-19 patients. On the contrary, no such conclusion can be made with regard to the pediatric population according to our study since no cases of death cases were observed at our Institute, even though these parameters were significantly higher in children with SARS-CoV-2 infection.^26^

Hyponatremia and hypochloremia are not infrequent laboratory and clinical findings in infectious diseases. These may be present due to infection-induced hyperglycemia (hypertonic hyponatremia and hypochloremia), infection-induced hyperproteinemia (isotonic hyponatremia and hypochloremia) or infection-induced edema (hypotonic hyponatremia and hypochloremia).^9^

Elevated aminotransferase levels are a common biochemical abnormality in COVID-19 patients, most probably due to liver injury associated with the immune response. However, except in the sense of being another diagnostic marker, their prognostic significance still remains uncertain.^10^

The serum concentration of LDH is an important marker of tissue damage and its elevation has been correlated with worse outcomes in cases of viral infections in general.^11^ Moreover, because of inflammatory reactions and tissue destruction, CRP levels in SARS-CoV-2-positive children tend to increase significantly.^27^

This study had some limitations. The first limitation was the small number of participants, given that this was a single-center study and that many children affected by the virus have no symptoms. Nonetheless, our findings could provide the basis for further research. The second limitation was that inflammation-related biomarkers, such as procalcitonin, interleukin-6 and presepsin were not included. Nor were hemostasis biomarkers such as partial thromboplastin clotting time (PTT), activated partial thromboplastin clotting time (aPTT), fibrinogen and D-dimer. Unfortunately, no such data were available for all the patients with confirmed SARS-CoV-2 infection because such analyses do not form part of the standard diagnostic protocol upon admission to our Institute, and are performed on demand only. Therefore, the baseline laboratory parameters were the main focus of interest in our study.

## CONCLUSION

All the laboratory markers mentioned above may help healthcare providers in early identification of COVID-19 patients. All of these parameters may be used for developing novel diagnostic scores for pediatric COVID-19 patients. CRP, AST and LDH demonstrated the best diagnostic performances, considering their sensitivity and specificity. Based on this study, it can additionally be concluded that healthy controls are superior to COVID-like controls since they provided better insight into the laboratory characteristics of children with SARS-CoV-2 infection.

## References

[1] Li Q, Guan X, Wu P (2020). Early Transmission Dynamics in Wuhan, China, of Novel Coronavirus-Infected Pneumonia. N Engl J Med..

[2] Worldometers COVID-19 coronavirus pandemic.

[3] Institute for Public Health Reports - Coronavirus COVID-19 https://covid19.rs/eng-instituteforpublichealth-updates/.

[4] Dhama K, Khan S, Tiwari R (2020). Coronavirus Disease 2019-COVID 19. Clin Microbiol Rev..

[5] Xu XW, Wu XX, Jiang XG (2020). Clinical findings in a group of patients infected with the 2019 novel coronavirus (SARS-Cov-2) outside of Wuhan, China: retrospective case series. BMJ.

[6] Henry BM, Lippi G, Plebani M (2020). Laboratory abnormalities in children with novel coronavirus disease 2019. Clin Chem Lab Med..

[7] Huang C, Wang Y, Li X (2020). Clinical features of patients infected with 2019 novel coronavirus in Wuhan, China. Lancet..

[8] Tiwari DN, Nath DD, Madan DJ (2020). Novel Insights into the Hematological Parameter Abnormalities in Pediatric COVID-19 Cases: Observation from A Preliminary Study of 11 Pediatric COVID-19 Cases in A Tertiary Care Center of North India. Saudi J Pathol Microbiol..

[9] De Carvalho H, Richard MC, Chouihed T (2021). Electrolyte imbalance in COVID-19 patients admitted to the Emergency Department: a case-control study. Intern Emerg Med..

[10] Moon AM, Barritt AS (2021). Elevated Liver Enzymes in Patients with COVID-19: Look, but Not Too Hard. Dig Dis Sci..

[11] Henry BM, Aggarwal G, Wong J (2020). Lactate dehydrogenase levels predict coronavirus disease 2019 (COVID-19) severity and mortality: A pooled analysis. Am J Emerg Med..

[12] Mardani R, Vasmehjani AA, Zali F (2020). Laboratory Parameters in Detection of COVID-19 Patients with Positive RT-PCR; a Diagnostic Accuracy Study. Arch Acad Emerg Med..

[13] Ali N (2020). Elevated level of C-reactive protein may be an early marker to predict risk for severity of COVID-19. J Med Virol..

[14] Ma H, Hu J, Tian J (2020). A single-center, retrospective study of COVID-19 features in children: a descriptive investigation. BMC Med..

[15] Soraya GV, Ulhaq ZS (2020). Crucial laboratory parameters in COVID-19 diagnosis and prognosis: An updated meta-analysis. Med Clin Barc..

[16] Garciá-Tardón N, Abbes AP, Gerrits A, Slingerland RJ, Den Besten G (2020). Laboratory parameters as predictors of mortality in COVID-19 patients on hospital admission. J Lab Med..

[17] Lippi G, Plebani M (2020). The critical role of laboratory medicine during coronavirus disease 2019 (COVID-19) and other viral outbreaks. Clin Chem Lab Med..

[18] Jiehao C, Jin X, Daojiong L (2020). A case series of children with 2019 novel coronavirus infection: Clinical and epidemiological features. Clin Infect Dis..

[19] Wang D, Ju XL, Xie F (2020). Clinical analysis of 31 cases of 2019 novel coronavirus infection in children from six provinces (autonomous region) of northern China. Chinese J Pediatr..

[20] Jung RG, Di Santo P, Clifford C (2021). Methodological quality of COVID-19 clinical research. Nat Commun..

[21] Yusuf E, Maiwald M (2021). COVID-19, equipoise and observational studies: a reminder of forgotten issues. Infection..

[22] Dettori JR, Norvell DC, Chapman JR (2019). Grab Control! Choosing the Right Comparison Group in an Observational Study. Glob Spine J..

[23] Goudouris ES (2021). Laboratory diagnosis of COVID-19. J Pediatr (Rio J)..

[24] Fathi N, Rezaei N (2020). Lymphopenia in COVID-19: Therapeutic opportunities. Cell Biol Int..

[25] Zheng SY, Xiao QY, Xie XH (2016). Association between secondary thrombocytosis and viral respiratory tract infections in children.. Sci Rep..

[26] Usul E, San I, Bekgöz B, Sahin A (2020). Role of hematological parameters in COVID-19 patients in the emergency room. Biomark Med..

[27] Wang L (2020). C-reactive protein levels in the early stage of COVID-19. Médecine Mal Infect..

